# Recovery trajectories of mental health symptoms among Chinese adolescents following the end of COVID-19

**DOI:** 10.3389/fpubh.2024.1396911

**Published:** 2024-12-04

**Authors:** Jun Peng, Meihui He, Yongxing Guo, Jiangdong Diao, Kun Chen, Ziyi Deng, Lei Mo, Ruixiang Gao

**Affiliations:** ^1^Center for Studies of Psychological Application, Guangdong Key Laboratory of Mental Health and Cognitive Science, and School of Psychology, South China Normal University, Guangzhou, China; ^2^DiggMind Psychometric Testing Technology Co., Guangzhou, China; ^3^Department of Psychology, School of Education Science, Hunan Normal University, Changsha, China; ^4^Nanjing Gulou District Education Bureau, Nanjing, China

**Keywords:** recovery trajectory, COVID-19, cumulative mental health risk, suicidal ideation, severity

## Abstract

**Background:**

The COVID-19 pandemic has had a profound impact on global mental health, particularly among adolescents. However, little is known about how mental health symptoms recover after the pandemic subsides. This study aims to examine the recovery trajectories of ten mental health problems and suicidal ideation among Chinese adolescents post-pandemic, with a focus on identifying factors that influence different recovery patterns.

**Methods:**

A total of 2,534 adolescents participated in a three-wave survey, conducted in June 2022, November 2022, and March 2023, using the Mental Health Scale for Chinese Middle School Students. A novel index was developed to account for both the number and severity of mental health risk factors, allowing for the classification of psychological symptoms into three subgroups: no, moderate, and severe. Polynomial regression models were applied to determine the acceleration inflection point, and multivariate logistic regressions identified predictors of trajectory membership.

**Results:**

Significant declines in psychological symptoms were observed. Seven trajectory patterns were identified: resistance (37.85%), recovery (22.61%), chronic-dysfunction (12.08%), aftermath-deterioration (10.81%), stress-responsiveness (8.21%), vulnerability (5.76%), and remitting (2.68%). Being female, senior high school students, and the oldest child in the family hindered mental health recovery, whereas parenting styles of companionship, empathetic support, promise fulfillment, and behavior intervention served as protective factors.

**Conclusion:**

This study is one of the first to reveal the post-pandemic mental health recovery trajectories of Chinese adolescents, highlighting the importance of considering both the number and severity of cumulative mental health problems. The findings offer valuable insights into suicide prevention and the development of targeted interventions to support youth mental health recovery.

## Highlights

- Chinese youths’ post-pandemic mental health recovery trajectories were first studied.
- Both the number and severity of cumulative psychological symptoms were considered.
- Significant psychological improvements were noted.
- Inflection point in how mental health issues influenced suicidal ideation was found.
- Gender, grade, sibling status, and parenting styles were shown to have an impact.

## 1 Introduction

There are substantial evidence that COVID-19 and related control measures have had an alarmingly negative impact on global mental health (1), increasing depressive and anxiety symptoms (2–5), affecting sleep (6–8), and subsequently leading to suicidal ideation (9, 10). Mental health protection and suicide prevention, especially among adolescents, were once a major government priority during the pandemic.

Now, with the pandemic drawing to a close, there is a notable gap in research, as no studies have yet examined the recovery of individuals’ mental health. According to the Allostatic Load Theory (11–13), the heightened level of chronic stress created by the prolonged period of COVID-19 had resulted in wear and tear on the individual’s allostatic response system. The illness, the lockdown, the loss of jobs of family members, and a number of other factors had taken a heavy toll on people’s minds (14). Once the acute phase of the pandemic is over, the transition back to more normal life allows the disturbed, overtaxed physiological and psychological systems to restabilize and return to homeostasis. It is anticipated that many mental health symptoms, as well as suicidal ideation, will significantly decrease as the stressor of COVID-19 disappears. However, this hypothesis has not yet been supported by empirical evidence. The present investigation aims to fill this knowledge void by exploring the longitudinal trajectories of mental health symptoms among Chinese adolescents after the completion of COVID-19.

In addition to the unknown recuperation trajectories, this study also seeks to address several deficiencies in prior research on COVID-19-related mental health. First, The majority of previous studies have tended to focus exclusively on one or two psychiatric disorders, such as depression, anxiety, or insomnia, with relatively less exploration of a more comprehensive profile of various indices, including obsessive-compulsive disorder (OCD), paranoia, hostility, etc. (3, 8, 15–17). Our former study indicated a significant covariation among various mental health indicators, suggesting that individuals who demonstrate positivity on one index are likely to exhibit it on others as well. Nonetheless, the risk of psychological crisis associated with multiple positive indices appears to be more severe than that with a single positive indicator, particularly in terms of suicide vulnerability (10). Therefore, it is advisable to assess multiple psychological indicators as comprehensively as possible within a study. In this research, we adopted the Mental Health Scale for Chinese Middle School Students (MSSMHS), which consists of 60 items to assess ten common psychological issues, including OCD symptoms, paranoia, hostility, interpersonal sensitivity, depression, anxiety, learning stress, maladjustment, emotional instability, and psychological imbalance. Besides, the item #57 is often used to assess suicidal ideation. This inventory, developed by the renowned Chinese psychologist Professor Jisheng Wang, is tailored to the cultural characteristics and behavioral habits of Chinese adolescents, making it comparable favorably to The Symptom Checklist-90-Revised (SCL-90-R) developed by western scholars (18).

Secondly, regarding suicide, while the accumulation of multiple positive indicators poses significant risks, equally perilous is having one single but highly severe positive index, referred to as the “huge stone” effect. However, no research to date has examined both the severity and the number of risk factors (i.e., mental disorders) at the same time. For instance, Ma et al. (10) investigated the cumulative effects of mental health risks (i.e., acute stress, depression, anxiety, insomnia, and obsessive-compulsive symptoms) on suicidal ideation during the COVID-19 pandemic. They deemed the factor with Z-score ≥ 1 as positive and risky, which was coded as 1, while they regarded those with Z-score < 1 as negative and risk-free, which was coded as 0. This is a typical research approach of the cumulative risk (CR) model (19–26). Nevertheless, unlike contextual risks, the risk of mental health problems is determined not only by the number of risky factors but also by the severity of each factor. The CR approach, though accounting for the cumulative effect of multiple mental health problems by calculating the number of factors coded as 1, failed to consider the variations in the severity of risk factors, as they treated the factors with Z-scores ≥2 to be equally risky as those with 1 < Z-score < 2. In the current research, we intended to propose a novel calculation method that simultaneously takes both the cumulative effect and the “huge stone” effect into account. Specifically, factors with a Z-score < 1 are deemed negative and risk-free, and coded as 0. Conversely, factors with a Z-score ≥ 1 are considered positive and risky, and assigned a value equivalent to their Z-score reflecting the severity of the risk. By summing up the values of all factors, we can obtain information that represents both the severity and the number of risks. This innovative index of the cumulative Z-score of “risky” factors (CZ-score) may effectively address the limitations of extant CR literature, yielding more statistical power for suicide prediction. In this study, to evaluate whether our CZ-score surpasses Juster et al.’s (11) traditional CR approach (CR-score), which counts the number of risk factors, we planned to use *R*^2^ as a measure of statistical power. *R*^2^ represents the ability of an index to accurately detect significant associations between cumulative mental health risks and suicidal ideation, reducing the likelihood of Type II errors (i.e., failing to detect a true effect). If the *R*^2^ of our CZ-score exceeds that of the CR-score in explaining the impact of cumulative mental health risks on suicidal ideation, it would demonstrate that capturing both the quantity and severity of risks provides superior sensitivity and accuracy compared to traditional CR models.

Third, while adolescents with a CZ score ≥ 1 can be considered to have mental health problems, a more detailed classification of their mental health status is of great importance to identify those in crisis. Referring to the pioneering work of Ma et al. (10) in determining the cumulative effect of multiple mental health risk issues (i.e., depression, anxiety, insomnia, acute stress, and OCD symptoms) on suicidal ideation among college students in the pandemic, individuals with 3 or more mental health risk factors were significantly more likely to experience a sharp increase in suicidal ideation. However, they did not account for the severity of mental health problems. In this study, we went beyond Ma et al. (10) with the revised CZ-score index to redetermine the inflection point between cumulative mental health risk and suicidal ideation. To identify this inflection point, we planned to employ a third-order polynomial regression model, which allows us to fit a curve that can capture complex, non-linear relationships between cumulative mental health risks and suicidal ideation. A third-order polynomial can create an “S”-shaped curve with two changes in curvature, which makes it well-suited to modeling situations where risk factors may initially increase slowly, then accelerate, and finally level off or even decrease. To locate the inflection point, we would use the second derivative of the polynomial function. The second derivative represents the rate of change of the slope of the original function—essentially, it tells us how the “steepness” of the curve is changing. When we look at the graph of a third-order polynomial, the inflection point appears where the convexness of the curve changes, indicating a significant change in the rate at which suicidal ideation risk is increasing. In simpler terms, the second derivative helps identify the inflection point, where cumulative risks start to accelerate sharply, marking a critical threshold beyond which the impact of mental health problems becomes significantly more severe. This point on the graph is crucial for distinguishing between moderate and severe psychological symptoms, thereby allowing us to identify individuals who are at a higher risk of a psychological crisis and need targeted intervention. Based on this inflection point, we intended to further refine the cut-off value of the CZ-score to distinguish between moderate and sever psychological distresses, dividing participants into three subgroups: one with a CZ-score < 1, indicating no psychological symptoms; one with a 1 ≤ CZ-score < the inflection point, indicating moderate psychological symptoms but no/minimal crisis; and one with a CZ-score ≥ the inflection point, indicating severe psychological symptoms and potentially high crisis. Based on this classification, we set out to reveal the risk trajectory of cumulative mental health problems of Chinese adolescents during the last phrase of COVID-19 and associated factors.

In summary, the current study aims to investigate the post-pandemic recovery trajectories of Chinese adolescents’ mental health by utilizing a novel approach of CZ-score index to comprehensively assess the number and severity of psychological symptoms and thus to screen out those in crisis from those with mild issues. It carries important implications for supporting youth mental well-being now that acute pandemic threats have passed with potential psychological damages lingering.

This study is a three-wave online questionnaire survey conducted in a middle school in Hubei Province, China, during the late phase of the COVID-19 pandemic. The first wave took place in June 2022 (T1), the second wave in November 2022 (T2), and the third wave in March 2023 (T3). During the first two waves, standard epidemic prevention and control measures were in effect, reflecting the ongoing challenges posed by the pandemic. Specifically, compared to T1, the epidemiological situation at T2 was more severe. However, by the time of the third wave, the situation had significantly improved after a challenging period of widespread infections resulting from the complete easing of pandemic restrictions during December 2022–January 2023, and the survey was conducted under the conditions of complete relaxation of epidemic control measures.

Based on the trajectory of the pandemic and the insights from previous literature on the trajectory of disaster-related mental health issues (4, 5, 9, 27), we expected to identify seven transition cohorts: (1) Resistance: minimal or no symptoms over time; (2) Chronic-dysfunction: moderate to severe symptoms persisting over time; (3) Recovery: initially moderate to severe symptoms followed by a gradual return to normal functioning; (4) Remitting: subsiding of symptoms but without full recovery to a healthy level; (5) Stress-responsiveness: mental health fluctuating in response to the severity of the pandemic—intensifying during peak periods and alleviating during downturns; (6) Vulnerability: in comparison to “Stress-responsiveness,” difficulty in timely relief of exacerbated psychological issues during the peak of the pandemic; (7) Aftermath-deterioration: delayed dysfunction manifesting only after the conclusion of the pandemic. The focal point of our investigation centers on the comparison of five pairs of cohorts. First, the contrast between the resistance cohort and the non-resistance cohorts (comprising the other six cohorts) aims to unveil protective factors that uphold adolescents’ psychological well-being throughout the pandemic. Second, examining the chronic-dysfunction cohort alongside the recovery and remitting cohorts aims to shed light on elements facilitating mental health improvement post the COVID-19 era. Third, a further nuanced comparison between the recovery cohort and the remitting cohort seeks to explain why some participants struggle to fully recuperate to a healthy level. Moving on, the juxtaposition of the stress-responsiveness cohort and the vulnerability cohort unveils factors that impede certain adolescents from timely alleviation of heightened psychological symptoms resulted from the pandemic peak. Finally, scrutinizing the aftermath-deterioration cohort against the resistance cohort aims to unravel the reasons behind some adolescents experiencing an anomalous pattern of changes deviating from the overall pandemic situation. While the first two comparisons were frequently made in previous research on symptoms entering the pandemic (4, 5, 9), the significance of the other three comparisons typically lies in the context of the pandemic’s exit.

As for the influencing factors that predict these seven cohorts, our study primarily concentrated on the family environment, along with considerations for gender and grade, because family attributes have been proven to play an important role in the psychological well-being and adjustment of Chinese adolescents (28–35). The factors of interest encompassed: the residency (i.e., urban or rural), sibling status (i.e., being the only child or not) and birth order among siblings, co-residence with parents (i.e., being a left-behind child or not), as well as parenting styles including considerations such as providing companionship to the child, granting autonomy, offering empathetic support, keeping promises made to the child, and intervening in inappropriate behaviors. Investigating these influencing factors provides valuable insights into supporting the post-pandemic psychological recovery of Chinese adolescents, tailoring effective strategies to foster mental well-being in the aftermath of COVID-19.

## 2 Methods

### 2.1 Participants and procedure

The study utilized a longitudinal design. A total of 2,534 middle school students (1,215 girls) from Jingzhou, China completed three waves of the mental health survey. At T1, 862 of the participants were in their second year of junior high (8th grade), 752 in their first year of senior high (10th grade), and 920 in their second year of senior high (11th grade). By T2, these students had advanced to 9th, 11th, and 12th grades, respectively. In the following sections, the grade at T1 was used to represent the corresponding group and referred to as Grade 8, 10, or 11.

Prior to data collection, we performed a power analysis using G*Power 3.1 to determine the sample size. For the polynomial regression analysis planned in this research, the minimum required sample size was calculated to be 43, assuming an alpha error probability of 0.05 and a power of 0.80. This ensures that our sample size (*N* = 2,534) was adequate to detect significant effects.

The data was collected from systematic surveys of student mental health status conducted on a semester basis. The local education bureau orchestrated the surveys, and the schools administered them. All participants provided electronic informed consent before beginning the online survey. Data were collected and managed through the DiggMind platform,^1^ a homegrown platform designed to provide psychological assessment services for schools across the country. The list of names of the participants found to be in crisis was reported to the school. Appropriate protocols were followed to secure permission to access and use the anonymous data for research purposes. This study was approved by the Biomedical Research Ethics Committee, Hunan Normal University (protocol number: 2021–411; date of approval: 13 May 2021).

### 2.2 Measures

The MSSMHS (see the supplementary file for its items) was administered to participants to assess a total of 11 mental health indicators. Each of its ten subscales consists of six specific items that are scored on a 5-point Likert scale ranging from 0 (none) to 4 (severe). A mean score for each subscale was calculated for analysis. In this study, Cronbach’s *α* values for the ten subscales at three time points, respectively, were as follows: OCD symptoms (0.75, 0.80, and 0.82), paranoia (0.88, 0.90, and 0.91), hostility (0.88, 0.90, and 0.91), interpersonal sensitivity (0.84, 0.87, and 0.88), depression (0.88, 0.90, and 0.90), anxiety (0.92, 0.93, and 0.94), learning stress (0.89, 0.91, and 0.91), maladjustment (0.83, 0.86, and 0.87), emotional instability (0.85, 0.88, and 0.89), and psychological imbalance (0.81,0.86, and 0.87). Suicidal ideation was assessed with a single item (#57) on the MSSMHS. This scale was administered three times.

Besides, associated family factors were assessed and coded as follows: residency (1 = urban; 2 = rural), sibling status (1 = only child; 2 = oldest child; 3 = younger child), the guardians to live with (2 = with both parents; 1 = with single parent; 0 = with other relatives), companionship (0 = rarely; 1 = occasionally; 2 = often), autonomy granting (0 = rarely; 1 = occasionally; 2 = often), empathetic support (0 = rarely; 1 = occasionally; 2 = often), promise fulfillment (0 = rarely; 1 = occasionally; 2 = often), and behavior intervention (0 = rarely; 1 = occasionally; 2 = often). These variables were measured once at T1 with single items, and were dummy-coded, utilizing the first value as the reference (ref.), when entering the logistic regression analysis.

### 2.3 Data analysis

Data analysis consisted of four main steps. Firstly, the mean with standard deviation and the prevalence rate (indicated by a Z-score ≥ 1) for the 11 indicators at three time points were each depicted. Of note, the Z-score was calculated based on the normative mean and standard deviation derived from a population estimate of over 100,000 students assessed through the DiggMind platform, rather than the mean and standard deviation of the current sample. This statistical approach is more sound and robust because, theoretically, even when samples are drawn from exceptionally healthy populations, a certain proportion of individuals may exhibit psychological disorders if the sample mean and standard deviation are employed.

Secondly, the CZ-score index was calculated by summing up the Z-score of “risky” factor indicated by a Z-score ≥ 1, and the crisis screening cut-off value of the CZ-score was subsequently determined. At this phase, we first compared the statistical power (*R*^2^) of our CZ-score in predicting suicidal ideation with Ma et al.’s (10) traditional CR approach of counting the number of risk factors (CR-score) in an effort to validate our novel index. Then, we employed a third-order polynomial regression model to identify the inflection point between cumulative mental health risk and suicidal ideation by examining its second derivative. In mathematical terms, the solution to the second derivative represent the position of knee point on the curve, on either side of which the concavity is different.

Thirdly, based on the cut-off point determined above, three subgroups of no, moderate, and severe psychological symptoms were classified at each time point, and different patterns of psychological symptom trajectories were then established based on time-varying changes of the subgroups. We expected to detect seven trajectory patterns: resistance, chronic-dysfunction, recovery, remitting, stress-responsiveness, vulnerability, and aftermath-deterioration.

Finally, five multivariate logistic regressions were used to examine predictors of mental health recovery cohorts. Our major interest lies in five comparisons, utilizing the former group as ref.: (1) non-resistance (encompassing all other cohorts except resistance) versus (vs.) resistance; (2) chronic-dysfunction vs. improvement (including recovery and remitting); (3) recovery vs. remitting; (4) stress-responsiveness vs. vulnerability; and (5) resistance vs. aftermath-deterioration.

## 3 Results

### 3.1 Mental health status across time

**Figure 1:**
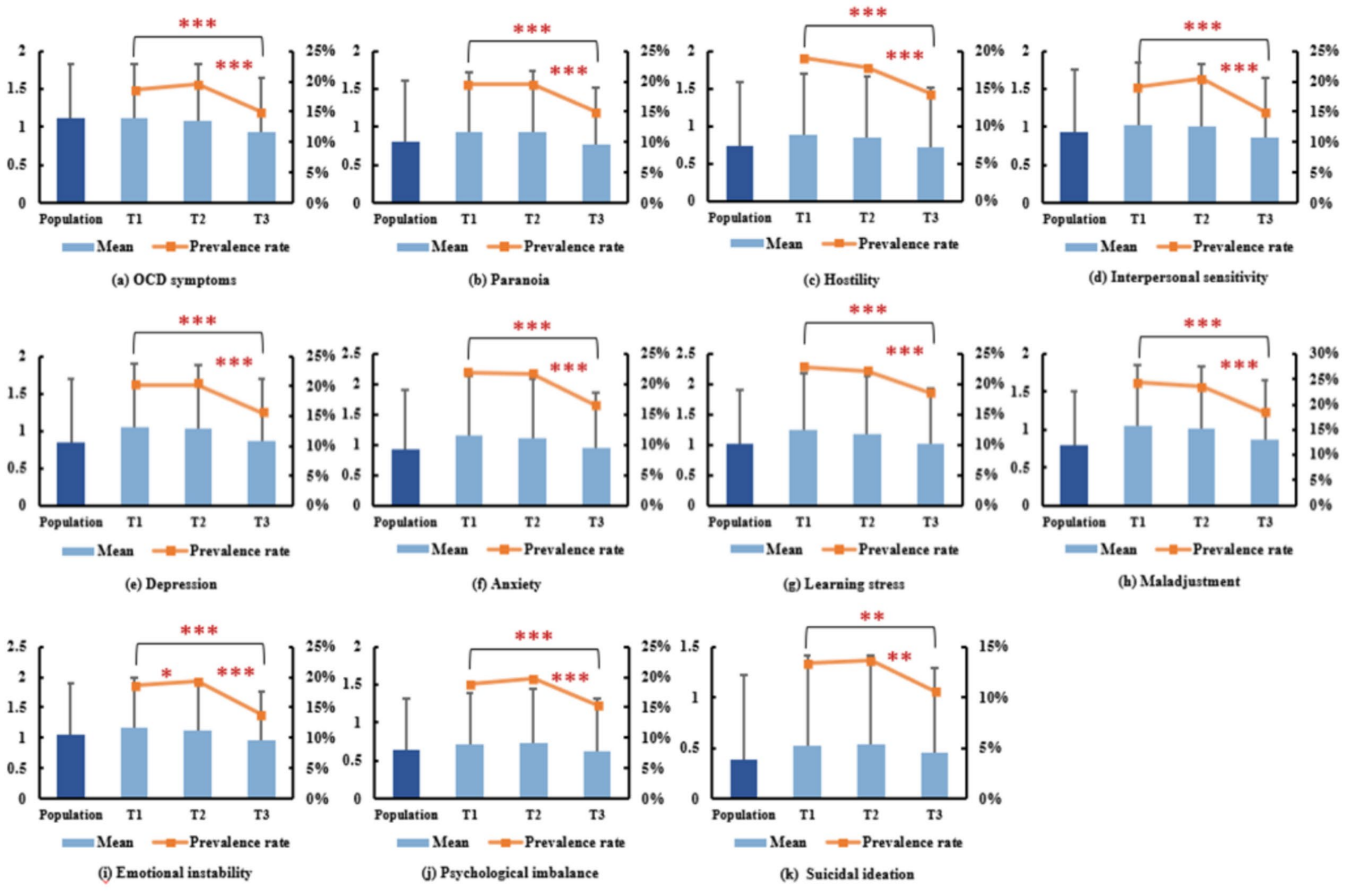
Overall status of the 11 mental health indicators at three time points. Note: The error bar denotes standard deviation. *p < 0.05, **p < 0.01, ***p < 0.001.

Figure 1 presents the sample’s mean, standard deviation, and prevalence (indicated by a Z-score ≥ 1) rate of the 10 psychological symptoms as well as suicidal ideation. Significant post-pandemic declines were observed in all 11 indicators, suggesting a favorable recovery trend.

### 3.2 Cut-off value for suicide screening

To validate our proposed CZ-score index, we first examined its associations with suicidal ideation using polynomial regression models, and compared these associations to those of the traditional CR-score index utilized by Ma et al. (10). Table 1 demonstrates that third-order polynomial fitting was generally applicable in revealing these associations. With consideration given to 10 mental health problems, our CR-score results achieved a statistical power of 36–42%, substantially higher than the maximum *R*^2^ of 15% reported by Ma et al. (10) for five mental health problems. However, upon adopting the novel CZ-score index, which accounts for not only the number but also the severity of mental health problems, our *R*^2^ experienced a further improvement ranging from 2 to 11%. Specifically, the more severe the pandemic situation (T2), the greater the increase in *R*^2^. This affirms the necessity and advantages of our CZ-score index.

**Table 1:** The association between cumulative mental health problems and suicidal ideation using polynomial regression models.

			Suicidal ideation (T1) as *Y*				Suicidal ideation (T2) as *Y*				Suicidal ideation (T3) as *Y*		
			*β*	*F* (*df*)	*R* ^2^		*β*	*F* (*df*)	*R* ^2^		*β*	*F* (*df*)	*R* ^2^
Linear model	*X*	CZ-score (T1) as *X*	0.613***	1528.075 (1)***	0.376	CZ-score (T2) as *X*	0.718***	2696.154 (1)***	0.516	CZ-score (T3) as *X*	0.665***	2009.832 (1)***	0.443
Quadratic model	*X*		0.738***	773.554 (2)***	0.379		0.726***	1347.610 (2)***	0.516		0.796***	1019.609 (2)***	0.446
	*X* ^2^		−0.136***				−0.009				−0.144***		
Cubic model	*X*		1.050***	528.474 (3)***	0.385		0.812***	899.439 (3)***	0.516		0.661***	682.022 (3)***	0.447
	*X* ^2^		−0.902***				−0.218				0.188		
	*X* ^3^		0.503***				0.137				−0.219*		
Linear model	*X*	CR-score (T1) as *X*	0.600***	1420.607 (1)***	0.359	CR-score (T2) as *X*	0.643***	1787.389 (1)***	0.414	CR-score (T3) as *X*	0.649***	1839.089 (1)***	0.421
Quadratic model	*X*		0.606***	710.036 (2)***	0.359		0.433***	905.467 (2)***	0.417		0.498***	925.345 (2)***	0.422
	*X* ^2^		−0.007				0.218***				0.156**		
Cubic model	*X*		0.820***	475.310 (3)***	0.360		0.954***	615.255 (3)***	0.422		0.873***	623.057 (3)***	0.425
	*X* ^2^		−0.627*				−1.289***				−0.956**		
	*X* ^3^		0.421*				1.016***				0.757***		

β is standardized regression coefficient. The CZ-score is the our proposed index that accounts for both the number and severity of mental health problems simultaneously. The CR-score is a traditional index used by Ma et al. (10) that accounts for only the number of mental health problems. *p < 0.05, **p < 0.01, ***p < 0.001.

Subsequently, we took the second derivative of the T1 cubic model of the CZ-score to determine the cut-off value—a critical acceleration point in the associations—that separates those in psychological crisis from those experiencing moderate psychological distress. As a result, the CZ-score = 16.36 was identified as the inflection point, after which the impact of cumulative mental health problems on suicidal ideation accelerated. The corresponding suicidal ideation score and Z-score at T1 were 1.59 and 1.44, respectively. Therefore, we were able to categorize three intervals in the CZ-score, i.e., 0–1, 1–16.36, and greater than 16.36, representing no, moderate, and severe psychological symptoms, respectively. The prevalence rates for severe psychological symptoms were 6.00% (T1), 6.67% (T2), and 3.79% (T3).

### 3.3 Recovery trajectory of psychological symptoms

**Figure 2:**
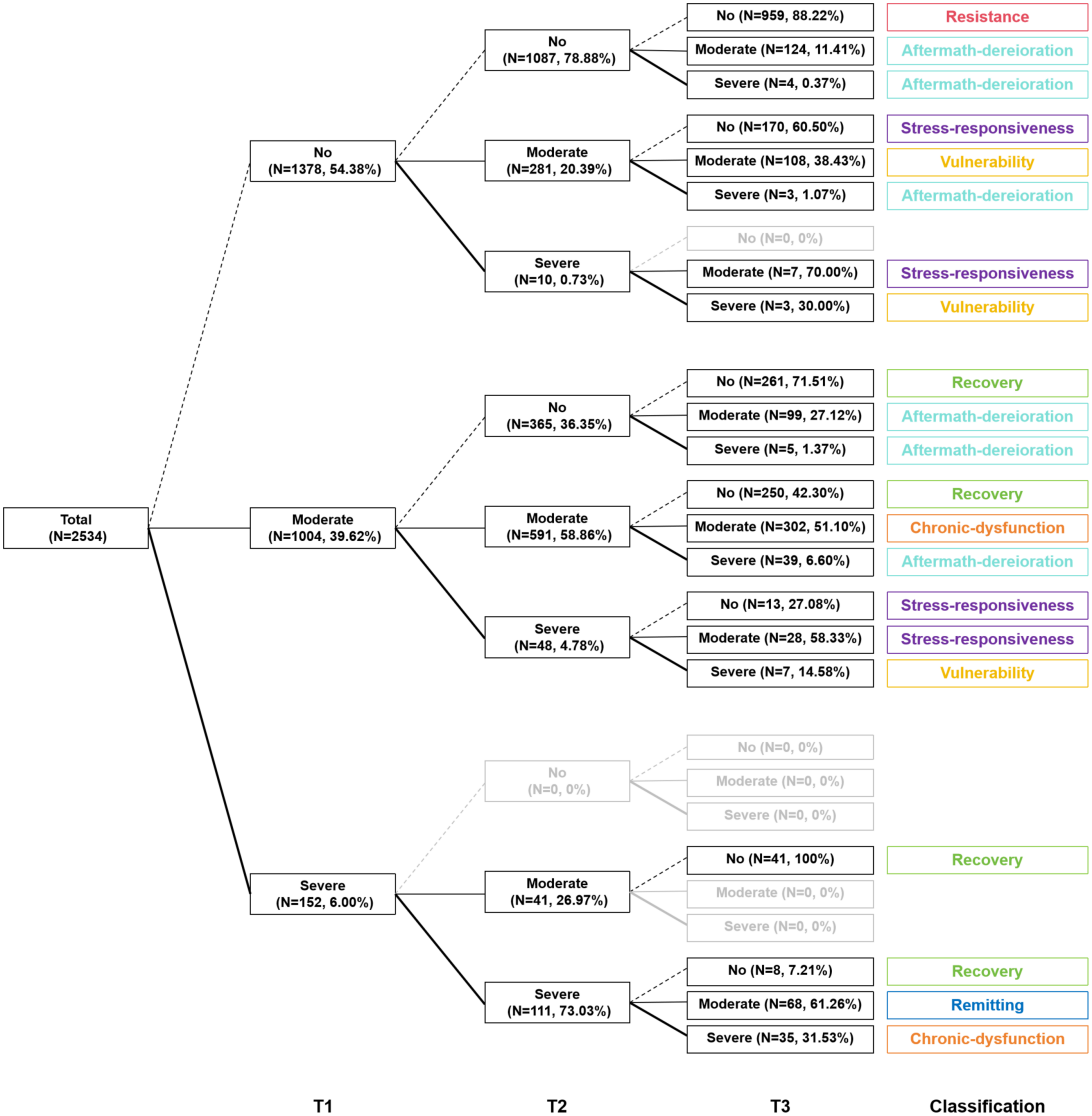
Change patterns of psychological symptoms.

**Figure 3:**
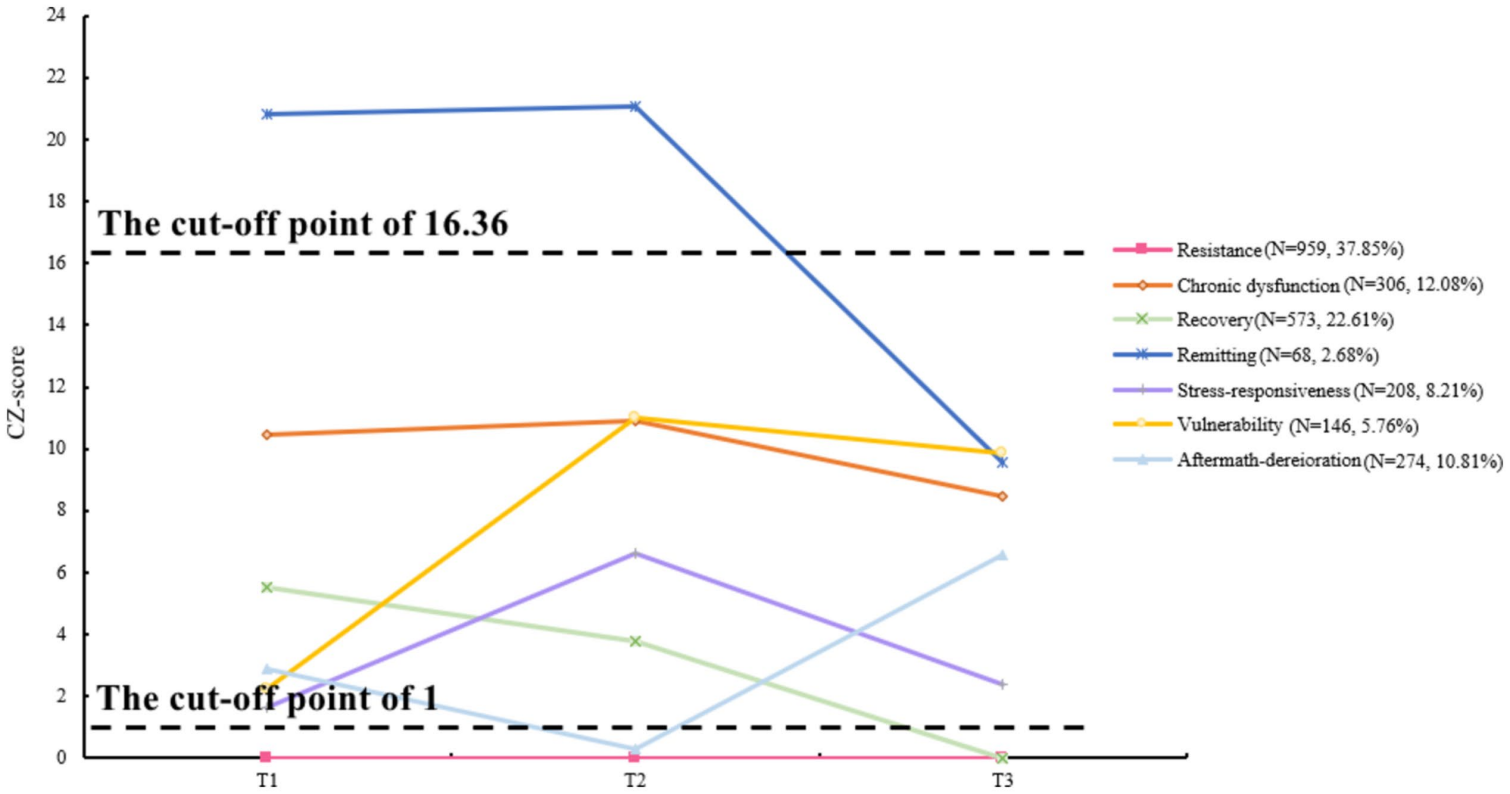
Trajectories of psychological symptoms.

After successfully classifying each participant into one of the three subgroups at each time point, we mapped how different subgroups of participants’ psychological symptoms changed over time and presented the trajectories depicted in Figures 2, 3. Among the seven transition cohorts, resistance had the highest proportion, followed by recovery, both of which, along with the smallest cohort of remitting, accounted for more than 60% of the sample, indicating an overall favorable mental health status. However, it was not without any challenges. The third-largest cohort (chronic-dysfunction) indicated that one in eight adolescents’ mental health issues truly warranted our concern, as they consistently faced psychological distress across three time points. Besides, surprisingly, about 10% of adolescents (aftermath-deterioration) experienced delayed dysfunction after the conclusion of COVID-19. This anomalous trajectory, deviating from the general pattern of pandemic changes, also demanded our consideration. Moreover, approximately 14% of the sample (stress-responsiveness and vulnerability cohorts) were significantly affected by the high peak of the epidemiological situation at T2, but two-fifths of them (vulnerability) did not well recuperate despite the easing of the situation, which called for attention as well. In total, around 30% of the participants might need psychological intervention.

### 3.4 Predictors of transition cohorts

At last, we examined the factors associated with an increased likelihood of resulting in the mental health conditions of interest using multivariate logistic regressions, and the results are shown in Table 2. First, being female (vs. male) or attending senior high schools (vs. junior high schools) was associated with poorer mental health, leading to decreased odds in the resistance cohort relative to the nonresistance cohort, increased odds in the remitting cohort relative to the recovery cohort, and increased odds in the aftermath-deterioration cohort relative to the resistance cohort. These results suggest that more attention should be paid to female adolescents and seniors. Secondly, being the oldest child in a family with more than one child, as opposed to being the only child, also impeded psychological well-being, leading to a decreased likelihood in the recovery cohort compared to the non-resistance cohort and a increased likelihood in the aftermath-deterioration cohort than the resistance cohort. It indicated that the oldest child in the family might bear more pressure. Thirdly, four out of the five examined parenting styles demonstrated protective roles. Adolescents whose parents accompanied them, provided empathetic support, and fulfilled promises made to them were more likely to develop psychological resilience. Good companionship also reduced the likelihood of merely staying at symptom relief without complete recovery and the likelihood of experiencing aftermath-deterioration. Parents’ empathetic support decreased the child’s likelihood of developing aftermath-deterioration as well. Promise fulfillment increased the likelihood of being in the improvement cohort in comparison to the chronic-dysfunction cohort, as did behavioral intervention. These findings underscored the importance of parenting styles in maintaining adolescents’ mental well-being. However, no significant predictors were shown to influence the chance of being in the vulnerability cohort vs. the stress-responsiveness cohort, leaving room for future research.

**Table 2:** Predictors of mental health trajectory memberships.

		Non-resistance (as ref.) vs. Resistance		Chronic-dysfunction (as ref.) vs. Improvement		Recovery (as ref.) vs. Remitting		Stress-responsiveness (as ref.) vs. Vulnerability		Resistance (as ref.) vs. Aftermath-deterioration	
		B (SE)	OR (95% CI)	B (SE)	OR (95% CI)	B (SE)	OR (95% CI)	B (SE)	OR (95% CI)	B (SE)	OR (95% CI)
Gender (Boy as ref.)	Girl	−0.41(0.09)***	0.66(0.55,0.79)	−0.07(0.15)	0.93(0.70,1.23)	0.75(0.30)*	2.11(1.18,3.77)	0.06(0.23)	1.06(0.68,1.66)	0.21(0.15)	1.23(0.92,1.65)
Grade (Grade 8 as ref.)	Grade 10	−0.78(0.12)***	0.46(0.37,0.58)	−0.35(0.20)†	0.71(0.48,1.04)	0.24(0.45)	1.27(0.53,3.08)	0.27(0.31)	1.31(0.71,2.43)	0.47(0.19)*	1.60(1.11,2.31)
	Grade 11	−0.73(0.11)***	0.48(0.39,0.60)	−0.27(0.19)	0.76(0.52,1.12)	0.90(0.42)*	2.46(1.09,5.56)	−0.06(0.29)	0.94(0.53,1.67)	0.33(0.18)†	1.39(0.98,1.99)
Residency (Urban as ref.)	Rural	0.07(0.10)	1.07(0.88,1.30)	0.03(0.15)	1.03(0.76,1.39)	0.29(0.31)	1.34(0.73,2.46)	0.18(0.24)	1.19(0.74,1.93)	−0.15(0.16)	0.87(0.63,1.18)
Sibling status (Only child as ref.)	Oldest child	−0.21(0.11)†	0.81(0.65,1.00)	0.04(0.17)	1.04(0.74,1.47)	−0.37(0.34)	0.69(0.35,1.35)	0.11(0.26)	1.12(0.67,1.87)	0.60(0.17)***	**1.82(1.31,2.54)**
	Younger child	−0.20(0.13)	0.82(0.64,1.05)	−0.09(0.19)	0.92(0.63,1.33)	−0.52(0.43)	0.60(0.26,1.39)	−0.02(0.32)	0.98(0.53,1.84)	0.19(0.21)	1.21(0.81,1.82)
Guardian to live with (With both parents as ref.)	With single parent	−0.22(0.16)	0.80(0.58,1.10)	−0.16(0.23)	0.86(0.55,1.34)	0.09(0.45)	1.09(0.45,2.63)	0.21(0.39)	1.23(0.57,2.64)	0.27(0.25)	1.31(0.80,2.14)
	With other relatives	−0.04(0.13)	0.96(0.74,1.23)	−0.02(0.19)	0.98(0.67,1.43)	0.21(0.36)	1.23(0.60,2.51)	0.22(0.34)	1.24(0.64,2.41)	−0.09(0.21)	0.91(0.61,1.36)
Companionship (Rarely as ref.)	Occasionally	0.15(0.19)	1.16(0.80,1.67)	0.11(0.21)	1.11(0.73,1.68)	−0.27(0.37)	0.77(0.37,1.59)	−0.34(0.43)	0.72(0.31,1.65)	−0.20(0.28)	0.82(0.47,1.43)
	Often	0.58(0.19)**	1.79(1.23,2.60)	−0.10(0.25)	0.91(0.56,1.46)	−1.14(0.50)*	0.32(0.12,0.84)	−0.30(0.46)	0.74(0.30,1.82)	−0.60(0.29)*	0.55(0.31,0.96)
Autonomy granting (Rarely as ref.)	Occasionally	0.15(0.15)	1.16(0.86,1.56)	0.14(0.20)	1.15(0.78,1.71)	0.11(0.41)	1.11(0.50,2.50)	0.05(0.40)	1.05(0.48,2.32)	0.41(0.27)	1.51(0.89,2.55)
	Often	−0.03(0.16)	0.97(0.71,1.32)	0.00(0.21)	1.00(0.66,1.52)	0.60(0.43)	1.82(0.79,4.19)	0.69(0.42)	1.99(0.87,4.53)	0.39(0.28)	1.48(0.86,2.54)
Empathetic support (Rarely as ref.)	Occasionally	0.43(0.22)*	1.53(1.00,2.34)	0.18(0.21)	1.20(0.79,1.82)	0.26(0.39)	1.30(0.60,2.79)	0.54(0.44)	1.71(0.72,4.08)	−0.68(0.32)*	0.51(0.27,0.94)
	Often	1.22(0.22)***	**3.40(2.22,5.21)**	0.28(0.25)	1.32(0.82,2.14)	−0.74(0.50)	0.48(0.18,1.28)	−0.08(0.47)	0.92(0.37,2.33)	−1.36(0.32)***	0.26(0.14,0.48)
Promise fulfillment (Rarely as ref.)	Occasionally	0.43(0.21)*	1.54(1.01,2.34)	0.25(0.20)	1.28(0.86,1.90)	−0.77(0.37)*	0.47(0.23,0.95)	−0.77(0.55)	0.46(0.16,1.35)	0.27(0.36)	1.32(0.66,2.64)
	Often	0.81(0.22)***	**2.24(1.47,3.43)**	0.49(0.22)*	1.64(1.06,2.54)	−0.65(0.42)	0.53(0.23,1.20)	−0.98(0.59)†	0.38(0.12,1.19)	0.00(0.37)	1.00(0.49,2.05)
Behavior intervention (Rarely as ref.)	Occasionally	−0.06(0.19)	0.95(0.65,1.38)	0.58(0.27)*	1.79(1.05,3.05)	0.03(0.52)	1.03(0.37,2.87)	−0.05(0.47)	0.95(0.38,2.38)	−0.21(0.30)	0.81(0.45,1.47)
	Often	−0.11(0.18)	0.90(0.63,1.29)	0.48(0.26)†	1.62(0.98,2.66)	−0.91(0.52)†	0.40(0.14,1.12)	0.00(0.45)	1.00(0.41,2.43)	0.08(0.29)	1.09(0.62,1.91)
McFadden *R*^2^		0.127		0.019		0.144		0.036		0.083	

CI, confidence interval. Non-resistance included all other cohorts except the resistance cohort. Improvement included the recovery and remitting cohorts. Pandemic-prone included all other cohorts except the aftermath-deterioration cohort. ^†^*p* < 0.1, *p < 0.05, **p < 0.01, ***p < 0.001. Bold: *p* < 0.001 and OR > 1.5 were considered to have scientific and public health significance.

## 4 Discussion

The COVID-19 pandemic has caused widespread and long-lasting mental health challenges, especially among adolescents. While many studies have focused on the immediate psychological impacts of the pandemic (1), few have investigated how mental health symptoms evolve and recover once the acute phase of the crisis ends. Adolescents are a particularly vulnerable group due to their developmental stage, during which they are more susceptible to mental health issues such as anxiety, depression, and suicidal ideation (35). These issues can be exacerbated by the social isolation, educational disruptions, and heightened uncertainty caused by the pandemic (36, 37). Furthermore, China, with its unique cultural context and strict pandemic control measures, offers an important case study.

This study makes several key contributions to the literature. First and foremost, to the best of our knowledge, this is one of the first to reveal the mental health recovery trajectories of Chinese adolescents after the completion of COVID-19. The significance of this research lies in its focus on the recovery process, which provides essential insights into the long-term effects of the pandemic and offers a foundation for understanding how adolescents adapt once the primary stressors are removed. Given the unprecedented scale of the pandemic and its profound disruption to adolescents’ lives, understanding how and why recovery occurs—or fails to occur—is crucial for designing interventions that address long-term psychological harm. Through a three-wave longitudinal design, we captured clear trends in the recovery trajectories of adolescents’ mental health as pandemic-related stressors eased. Significant reductions in mental health symptoms, along with the predominance of resistance and recovery cohorts, indicated an overall positive status. These recovery patterns are directly linked to the pandemic’s unique and profound social and psychological impacts, rather than common stressors like academic pressure or family dynamics. The COVID-19 pandemic introduced unprecedented, widespread, and prolonged stressors that affected adolescents in ways that are unlikely to be replicated in other contexts. Specific challenges such as prolonged social isolation, disruptions in education, and concerns about family health created a distinct psychological burden. As pandemic-related stressors diminished, we observed significant mental health improvements, reinforcing the conclusion that these changes are specifically tied to the post-COVID-19 environment. Thus, we argue that the recovery trajectories identified in this study are unique to the post-pandemic period and are unlikely to emerge in settings without such extensive disruptions. The significant reduction in psychological symptoms observed over time reflects the easing of pandemic-specific stressors, aligning with the well-established Allostatic Load Theory and supporting the stressor-exit effects.

However, the silver lining was not without its own cloud. The not-so-small existence of chronic-dysfunction, aftermath-deterioration, and vulnerability cohorts, revealed by our study, suggested the need for tailored psychological intervention. For adolescents in the chronic-dysfunction cohort, a long-term, structured intervention plan is essential. Cognitive Behavioral Therapy, which has been proven effective in managing persistent mental health conditions, can serve as the core therapeutic approach (38, 39). Group therapy can also be beneficial, as it provides peer support and helps reduce feelings of isolation (40, 41). In the aftermath-deterioration cohort who exhibit delayed mental health issues post-pandemic, psychoeducation combined with early detection can be crucial (42). Schools and communities can offer resilience-building programs that focus on early identification of warning signs, helping students understand the impact of stress, and providing coping strategies before symptoms escalate (43). The interventions could incorporate mindfulness-based techniques, such as Mindfulness-Based Stress Reduction (44), which has been shown to be effective in managing anxiety and depression. For the vulnerability cohort, routine-based programs such as Stress Inoculation Training (45), which aims to prepare individuals for future stressors by teaching them problem-solving and relaxation techniques, can be effective. Regular follow-up and monitoring also ensure sustained progress across cohorts. Further predictor analyses shed light on the subgroups of female adolescents, high school seniors, and the oldest child in the family who may exhibit inferior recovery outcomes, and emphasized the protective roles of some parenting styles such as companionship, empathetic support, promise fulfillment, and behavior intervention. These findings align with studies exploring the impact of COVID-19 on adolescent mental health across different family structures and dynamics. Francisco et al. (46) reported that household size significantly influenced children’s moods during the early phase of the COVID-19 quarantine, as sibling interactions became crucial for psychological well-being when peer contact was limited. Cooper et al. (47) found that adolescents who experienced greater loneliness had significantly higher mental health symptoms during lockdown, while those with closer relationships with parents reported fewer symptoms and lower levels of loneliness. However, adolescents who spent more time texting others exhibited higher mental health difficulties. Research also demonstrated that positive family cohesion, flexibility, and communication were associated with better individual well-being, higher family quality of life, and reduced conflict during the COVID-19 pandemic (48, 49). Collectively, these studies emphasize the role of family mutual support in mitigating the impact of home confinement on adolescents during the pandemic (50). Our findings contribute to this literature by offering a more nuanced understanding of who may benefit more or less from such support. For example, being older or the oldest child in the family may assume more of a caregiver role rather than being recipients of care, depleting rather than replenishing their psychological resources, which could lead to slower recovery after the pandemic. This suggests that family-based interventions aimed at improving communication and transforming post-pandemic challenges into opportunities for psychological and familial growth (51) should be tailored to address the unique needs of different family members.

Most important of all, this study justified the necessity to simultaneously account for both the number and severity of cumulative mental health problems and developed a novel methodological approach of the CZ-score index summing the Z-scores of all risky factors greater than 1, which addressed the limitations of traditional CR models. In traditional CR models (10), mental health risks are typically measured as binary indicators (presence or absence of a condition), which only account for the number of risk factors. While this approach offers simplicity, it overlooks the severity of individual symptoms, which can vary significantly across participants. Our CZ-score advances this approach by taking into account not only whether a mental health risk is present but also its severity, measured by the Z-score of each mental health factor. The rationale for this index is grounded in research showing that the impact of mental health issues on outcomes like suicidal ideation is not merely cumulative but also influenced by the intensity of each condition (19, 21). For instance, while a participant with multiple mild symptoms may be at risk, an individual with fewer but more severe symptoms may be at even higher risk. The CZ-score addresses this by assigning a Z-score to each factor above a certain threshold (Z ≥ 1) and summing these scores, allowing us to capture both the quantity and severity of psychological issues. This approach provides a more nuanced and powerful tool for identifying individuals at risk of psychological crisis, such as suicidal ideation. In the present study, the CZ-score calculation of multiple mental health issues achieved a 2–11% increase in explanatory power (indicated by *R*^2^ values) in predicting suicidal ideation in comparison to the traditional CR method. This increase was higher in a period of heightened epidemiological situation at T2, suggesting that consideration of the severity of psychological symptoms during major crisis events remains particularly critical. This finding introduces a crucial nuance to the existing CR literature. With the CZ-score index, we were able to identify the acceleration inflection point in the associations between suicidal ideation and cumulative mental health risks using polynomial regression models, and then to separate those in potential psychological crisis from those with merely moderate psychological symptoms to provide a more subtle picture. This also represents a significant innovation in suicide risk screening methodology, offering a novel feasible approach for psychological crisis identification during crisis events. Therefore, the theoretical contributions and practical implications of this study are substantial.

Nevertheless, several limitations need to be considered in our study. For one, the study sample was restricted to one city of China, and the sampling method employed was convenience sampling. Therefore, the recovery patterns identified may not be fully representative of all Chinese adolescents. For the other, because we were not able to discriminate the likelihood of being in the stress-responsive cohort from the vulnerability cohort using our selected predictors, future studies should thoroughly examine a range of risk and protective factors. More detailed family functioning variables (52, 53) and positive youth development attributes (54) should receive research attention.

## 5 Conclusion

In conclusion, this study pioneered the investigation of the post-COVID-19 mental health recovery trajectories of Chinese adolescents and revealed considerable improvements. The novel methodological approach of simultaneously considering the number and severity of cumulative mental health risk factors paved the way for a breakthrough in suicide risk screening in major crisis events. The identification of some inferior recovery trajectories, such as chronic-dysfunction, aftermath-deterioration, and vulnerability, underscores the need for tailored interventions that address the specific psychological needs of these groups.

## Data Availability

The raw data supporting the conclusions of this article will be made available by the authors, without undue reservation.

## Footnotes

^1^http://digme.cn/
